# Comparative analysis of single-cell transcriptome reveals heterogeneity in the tumor microenvironment of lung adenocarcinoma and brain metastases

**DOI:** 10.1007/s12672-023-00784-2

**Published:** 2023-09-15

**Authors:** Jialu Liang, Ruihao Liang, Kai Lei, Jing Huang, Huayue Lin, Minghui Wang

**Affiliations:** 1 Guangdong Provincial Key Laboratory of Malignant Tumor Epigenetics and Gene Regulation, Sun Yat-sen Memorial Hospital, Sun Yat-sen University, Guangzhou, China; 2 Department of Thoracic Surgery, Sun Yat-sen Memorial Hospital, Sun Yat-sen University, Guangzhou, China; 3 Breast Tumor Center, Sun Yat-sen Memorial Hospital, Sun Yat-sen University, Guangzhou, China

**Keywords:** scRNA-seq, Lung adenocarcinoma, Brain metastasis, TME, DDIT4

## Abstract

**Purpose:**

Solid tumors such as lung adenocarcinoma include not only the tumor cells but also the microenvironment in which the tumor cells continuously interact with each other. An in-depth understanding of the oncological features and tumor microenvironment (TME) of lung adenocarcinoma and brain metastases at the single-cell level could provide new therapeutic strategies for brain metastases from lung adenocarcinoma.

**Methods:**

To solve this problem, we performed single-cell RNA sequencing (scRNA-seq) analysis on 15 lung adenocarcinoma samples and 10 brain metastasis samples.

**Results:**

A total of 86,282 single cells were obtained and divided into 8 cell types, including epithelial cells, endothelial cells, fibroblasts, oligodendrocytes, T/NK cells, B cells, mast cells, and macrophages. In brain metastases, we found a significantly lower proportion of T/NK cells and mast cells, and more severe immune dysregulation. In addition, we found a subpopulation of macrophages with high expression of metastasis-promoting-related genes enriched in brain metastatic tissues. Moreover, in brain metastases, we found a significantly increased proportion of myofibroblastic cancer-associated fibroblasts (myCAFs) and a higher angiogenic capacity of endothelial cells. Epithelial cells in brain metastases were more malignant and underwent genomic reprogramming. Next, we found that DNA damage-inducible transcript 4 (DDIT4) expression was upregulated in epithelial cells in brain metastases and was associated with poor prognosis. Finally, we experimentally validated that the downregulation of DDIT4 inhibited the proliferation, migration, and invasion of lung cancer cells.

**Conclusions:**

This study depicts a single-cell atlas of lung adenocarcinoma and brain metastases by scRNA-seq and paves the way for the development of future therapeutic targets for brain metastases from lung cancer.

**Supplementary Information:**

The online version contains supplementary material available at 10.1007/s12672-023-00784-2.

## Introduction

Lung cancer is one of the diseases with high morbidity and mortality rates worldwide, with adenocarcinoma being the most common pathological type of lung cancer [1]. Distant metastatic sites of lung cancer are commonly found in the brain [2]. Nearly 50% of lung cancer patients will develop brain metastases during the progression of the disease [3]. Lung cancer patients with brain metastases have a poor prognosis with a median survival of only 4–5 months [4]. Surgery plus radiotherapy is currently the main treatment for lung cancer patients with brain metastases, but the treatment effect is not ideal [5]. Therefore, an in-depth study on the mechanism of the development of brain metastases from lung cancer will be of great significance to prolong the survival time of patients and improve the quality of survival.

The tumor microenvironment (TME) refers to the non-tumor cells and components of the tumor, including the molecules produced and released by them [6]. The triad of immune response, extracellular mesenchymal remodeling, and tumor angiogenesis essentially determines the aggressiveness of tumors [7]. Therefore, understanding the integrated features of the tumor microenvironment can provide new therapeutic targets. In the highly complex tumor microenvironment, traditional transcriptomic sequencing has significant limitations in providing accurate information about individual cells [8]. Recent advances in scRNA-seq technologies provide a powerful tool to explore the heterogeneity of the tumor microenvironment [9]. Currently, there are several articles investigating the heterogeneity of lung cancer by scRNA-seq. For example, Lambrechts and colleagues provided a TME cell atlas of lung cancer by scRNA-seq, Guo and colleagues similarly used scRNA-seq to construct a single-cell atlas of T cells and found that a type of regulatory T cells (Tregs) was associated with poor prognosis in lung adenocarcinoma, and Zilionis et al. classified tumor-infiltrating myeloid cells, including monocytes, macrophages, dendritic cells, and granulocytes, into at least 25 different states by scRNA-seq [10–12]. However, few studies have explored the differences between lung adenocarcinoma and brain metastases at the single-cell level.

To bridge this gap, in this study, we first attempted to provide a single-cell atlas of lung adenocarcinoma and brain metastases. We analyzed single-cell transcriptomic data from 15 lung adenocarcinoma samples and 10 brain metastasis samples to present a comprehensive transcriptomic profile of lung adenocarcinoma and brain metastasis. We identified similarities and differences in the tumor cell and tumor microenvironment between lung adenocarcinoma and brain metastasis and elucidated that DDIT4 plays an important role in the process of lung cancer metastasis. This study can help explain the differences between lung adenocarcinoma and brain metastases by characterizing the cellular composition and status in the tumor microenvironment and pave the way for the development of future therapeutic targets for brain metastases from lung cancer.

## Methods

### Collection and processing of single-cell RNA sequencing data

We downloaded the scRNA-Seq dataset GSE131907 [13] from the Gene Expression Omnibus (GEO) database. A total of 25 samples were downloaded, including 15 lung adenocarcinoma samples and 10 brain metastasis samples. We removed cells that had less than 200 genes or contained more than 20% of the total expressed genes as a percentage of mitochondrial genes. A total of 57,222 lung adenocarcinoma tissue cells and 29,060 brain metastasis tissue cells were obtained. In addition, we removed double cells using the DoubletFinder (version 2.0.3) software package [14]. Considering the differences in processing operations between tumor tissues and brain metastases, we used Harmony [15] software (version 1.0) to eliminate batch effects between data sets. We continued to process the data using the Seurat [16] software (version 3.2.3). First, we normalized the combined data and performed the principal component analysis (dim = 25) on the top 2000 highly variable genes. Then, the cell nearest neighbor network was constructed using the FindNeighbors function, and finally, the cell clustering was performed using the FindClusters function (resolution = 0.1). Visualization was performed using the uniform manifold approximation and projection (UMAP) method. Differentially expressed genes were obtained using the “FindAllMarkers” or “FindMarkers” functions with default parameters. For subclustering analysis, we used the same approach to find highly variable genes, descending and clustering. All cell subgroups were manually annotated according to marker genes.

### Analysis of cell composition between patient groups

To assess whether there were significant differences in the cell type composition of patients in each group, we used the R package ggpubr for statistical testing and visualization. Comparisons between the two groups were statistically tested using the Wilcoxon rank-sum test. p-value < 0.05 were considered statistically different.

### Gene set variation analysis (GSVA)

We performed pathway analysis of 50 Hallmark pathways described in Molecular Signatures Database (MsigDB). To describe biologically important processes in specific cell types, we performed gene set enrichment analysis using the GSVA [17] package (version 1.38.0). The differential activity of pathways between different subgroups was calculated by the Limma [18] R package (version 3.46.0).

### Calculation of gene signature scores

To assess the potential function of the cell subpopulation of interest, we calculated the functional module scores of the cell subpopulation using Seurat’s AddModuleScore function.

Gene signatures were obtained from a previous study [11].

### Copy number variation (CNV) analysis

The 10X scRNA-seq data were analyzed using the inferCNV (version 1.6.0) method to illustrate the different patterns of chromosome copy number variation in tumor cells of different origins [19]. Here we randomly selected one-tenth of epithelial cells for analysis, with fibroblasts and endothelial cells as controls.

### Survival analysis of up-regulated genes in epithelial cells from brain metastases

We used TCGA lung adenocarcinoma and GSE68465 datasets to assess the prognostic value of up-regulated genes. We downloaded transcriptome expression and clinical data of 513 TCGA lung adenocarcinoma patients at UCSC Xena (http://xena.ucsc.edu/). In addition, we downloaded transcriptomic expression and clinical data of 442 lung adenocarcinoma patients from GSE68465. For survival analysis, all patients were divided into the high and low groups based on the mean of genes in these samples, and Kaplan-Meier curves were constructed to visualize the difference in overall survival (OS) between the high and low groups. p-value < 0.05 were considered statistically different.

### Cell culture and siRNA transfection

Two lung cancer cell lines (A549 and PC9) were purchased from Shanghai Institutes for Biological Sciences, China. A549 and PC9 were cultured in 1640 (Gibco, Carlsbad, CA, USA) medium supplemented with 10% fetal bovine serum (FBS) and 100 U/ml penicillin/streptomycin. Two cell lines were warmed at 37 °C and 5% CO2. Two cell lines were transfected with Liposome 3000 transfection reagent according to the manufacturer’s instructions. Control short interfering RNA (siRNA) and siRNA targeting DDIT4 were purchased from GenePharma (Shanghai, China). The siRNA sequences used for RNA interference analysis were as follows:5ʹ-GCUUCCGAGUCAUCAAGAAGATTUCUUCUUGAUGACUCGGAAGCTT-3ʹ. The control sequence was 5- UUCUCCGAACGUGUCACGUTTACGUGACGUUCGGAGAATT-3. The transfected cells were cultured in fresh medium for 48 h for subsequent assays.

### Real-time quantitative PCR

Total RNA was extracted from PC9 and A549 cells using TRIzol reagent and converted to cDNA using a reverse transcription kit (Vazyme, Nanjing, China). cDNA was then analyzed by RT-qPCR based on SYBR Green according to the manufacturer’s instructions. GAPDH was used as an internal control. The primers for DDIT4 are 5ʹ-TGAGGATGAACACTTGTGTGC-3ʹ (forward) and 5ʹ-CCAACTGGCTAGGCATCAGC-3ʹ (reverse), and GAPDH are 5ʹ-GGAGCGAGATCCCTCCAAAAAT-3ʹ (forward) and 5ʹ-GGCTGTTGTCATACTTCTCATGG-3ʹ (reverse).

### Cell proliferation assay

Cell Counting Kit-8 (CCK-8, Dojindo, Rockville, MD, USA) was used to assess cell proliferation according to the manufacturer’s instructions.

### Cell wound healing assay

Transfected lung cancer cells were cultured in 6-well plates containing 1 × 10^6^ cells/well for 48 h, and then scratches were generated with a 200 µL pipette tip. The images were taken of the scratch with a microscope at 0–48 h. The scratch area was measured with Image J software to evaluate the cell migration ability.

### Cell migration and invasion assays

Cell migration or invasion assays were performed with Corning Transwell Inserts (8.0 μm) inserted into 24-well plates with or without precoated diluted Matrigel (Becton Dickinson, Franklin Lakes, NJ, USA) using 10% FBS as chemoattractant. After 48 h (migration and invasion), the cells on the lower side of the membrane were fixed and stained by crystal violet. Finally, the stained cells were counted and photographed under an inverted microscope.

### Statistical analysis

All statistical analyses were performed using R 4.0.2 and GraphpadPrism 8.0 software. Data were presented as mean ± standard deviation (SD). The t-test (Mann-Whitney U test if necessary) was used to compare the differences between the two groups. p-value < 0.05 was statistically significant.

## Results

### Cell composition of tumor tissues and brain metastases

**Fig. 1:**
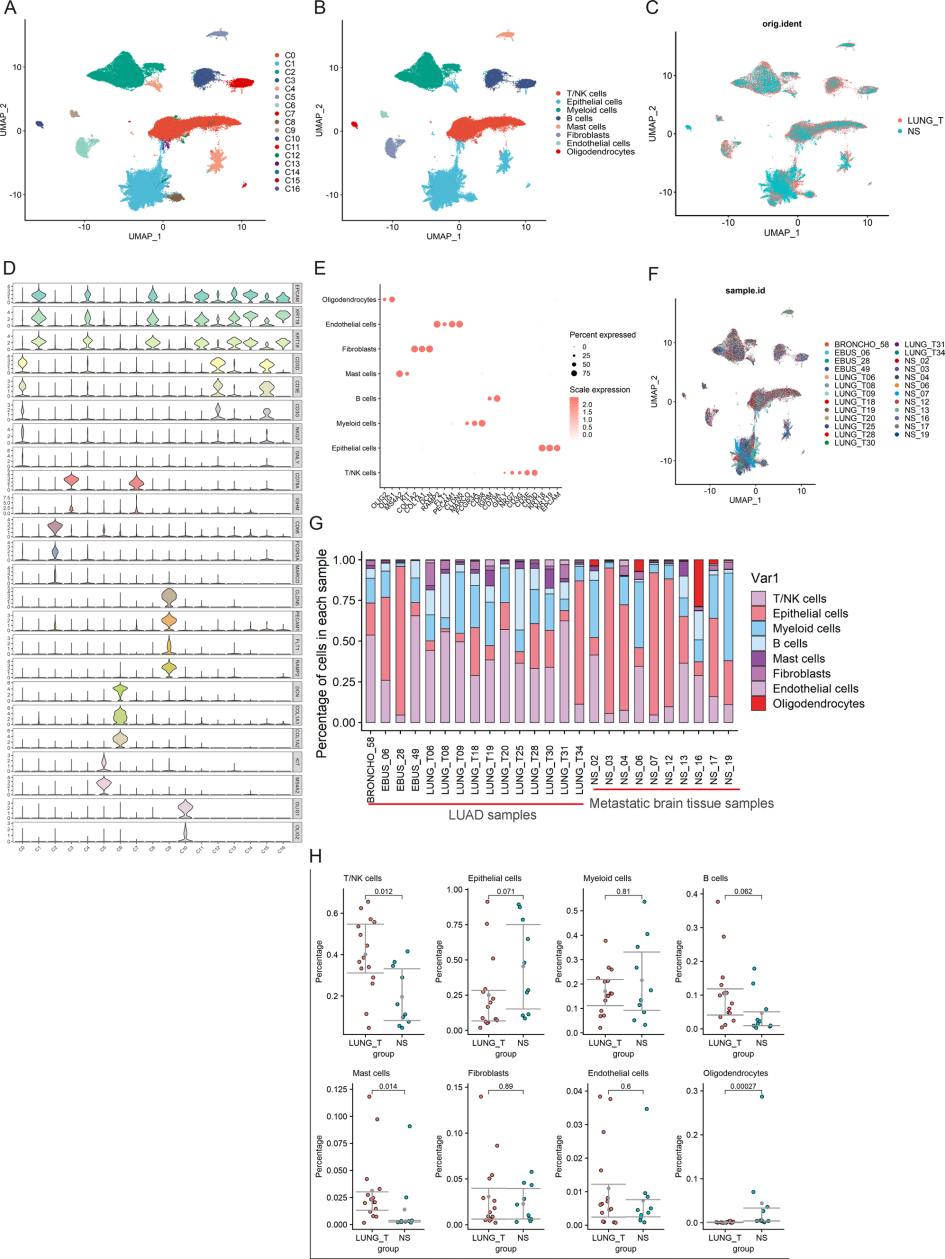
Overview of TME in lung adenocarcinoma and brain metastases. **A** UMAP visualization of all cells displayed with different colours for clusters. **B** Eight major cell types in lung adenocarcinoma and brain metastases. **C** Cell distribution in lung adenocarcinoma and brain metastases. **D** For violin plots, x-axes stand for the number of clusters, y-axes stand for the relative expression level of marker genes. **E** Marker genes of eight major cell types identified in this study. **F** The UMAP plot showing sample origin. **G** The proportion of each cell type in lung adenocarcinoma and brain metastases. **H** Percentages of the eight cell types among the two groups. Y-axis: average percentage of samples across the two groups. Groups are shown in different colours

After data processing, 57,222 cells from tumor tissues (n = 15) and 29,060 cells from brain metastases (n = 10) were obtained for subsequent analysis (Fig. 1C, F). All cells were divided into 17 subpopulations (Fig. 1A). We annotated each cell subpopulation according to the highly expressed marker genes (Fig. 1D). Eight cell types were identified in 17 subpopulations, including four non-immune cells and four immune cells (Fig. 1B). Non-immune cells included endothelial cells (CLDN5, PECAM1, FLT1, and RAMP2), fibroblasts (DCN, COL1A1, and COL1A2), epithelial cells (KRT18, KRT19, and EPCAM) and oligodendrocytes (OLIG2 and OLIG1) (Fig. 1E). Immune cells included macrophages (MARCO, CD68, and FCGR3A), mast cells (MS4A2, KIT), T/NK cells (GNLY, NKG7, CD3D, CD3E, and CD3D), and B cells (IGHM, CD79A) (Fig. 1E). Then, we calculated the proportion of each cell type in tumor tissues and brain metastases (Fig. 1G). We found that the proportion of T/NK cells and mast cells was significantly lower in brain metastases (Fig. 1H).

### Heterogeneity of NK/T cells in different lesions

**Fig. 2:**
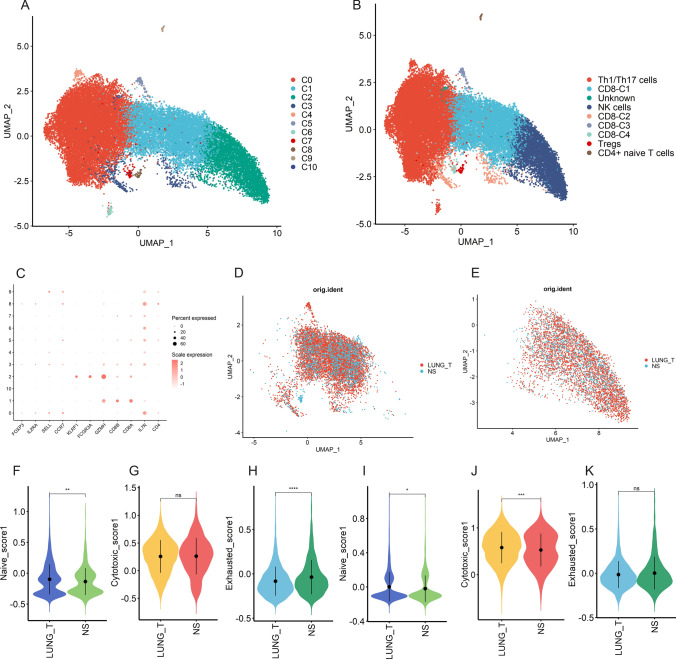
Heterogeneity of NK/T cells in lung adenocarcinoma and brain metastases. **A** UMAP plot shows eleven subclusters of the NK/T cells. **B** UMAP plot of NK/T cells revealing nine subtypes. **C** Canonical cell markers used to identify NK/T cell subtypes. **D** The UMAP plot showing CD8 + T cells origin. **E** The UMAP plot showing NK cells origin. **F**–**H** Violin plot indicating the naive (left), cytotoxic (middle), and exhausted (right) scores of CD8 + T cells from lung adenocarcinoma and brain metastases. **I**–**K** Violin plot indicating the naive (left), cytotoxic (middle), and exhausted (right) scores of NK cells from lung adenocarcinoma and brain metastases

We performed a subclustering analysis of 27,601 NK/T cells and obtained 11 cell subclusters (Fig. 2A). Four clusters (C1, C3, C5, and C7) exhibited high expression of CD8A and CD8B, defined as CD8 + T cells. Three clusters (C0, C4 and C6) defined as Th1/Th17 cells (CD8-, IL7R+). C2 with high expression of FCGR3A and KLRF12 was defined as NK cells. C8 with high expression of IL2RA and FOXP3 was defined as Tregs, and C9 with high expression of SELL and CCR7 was defined as CD4 + naive T cells. since no marker gene was found in the C10 subpopulation, we defined it as Unknown (Fig. 2B, C). To assess the potential functional and immune status of CD8 + T cells and NK cells, we used the AddModuleScore function to calculate the scores of functional modules of CD8 + T and NK cells from different sources (Fig. 2D, E). Notably, CD8 + T cells in brain metastases show lower naive scores and higher exhaustion scores compared to tumor tissues, while NK cells show lower naive scores and cytotoxicity scores (Fig. 2F–K).

### Heterogeneity of myeloid cells in different lesions

**Fig. 3:**
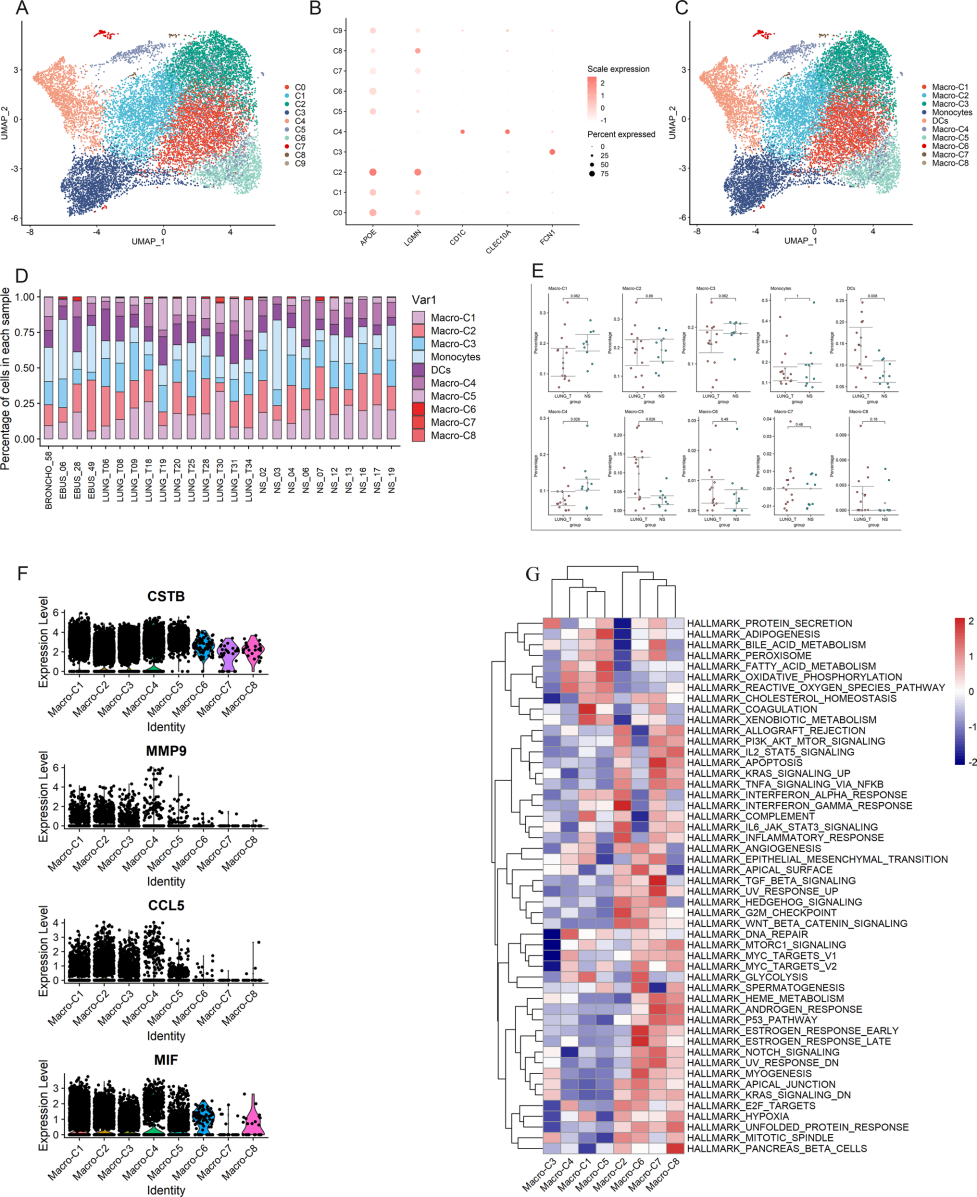
Heterogeneity of myeloid cells in lung adenocarcinoma and brain metastases. **A** UMAP plot shows ten subclusters of the myeloid cells. **B** Canonical cell markers used to identify myeloid cell subtypes. **C** UMAP plot of myeloid cells revealing ten subtypes. **D** The proportion of myeloid cell subtypes in lung adenocarcinoma and brain metastases. **E** Percentages of each myeloid cell subtype among lung adenocarcinoma and brain metastases. **F** Violin plots of the expression of several marker genes, including CSTB, MMP9, CCL5, and MIF, in macrophage subtypes. **G** Differences in pathway activities by GSVA among different macrophage subtypes

Next, we performed a subclustering analysis of all myeloid cells. A total of 10 cell subpopulations were obtained (Fig. 3A), including 8 clusters of macrophages (APOE, LGMN) (C0, C1, C2, C5, C6, C7, C8, C9), monocytes (C3) (FCN1) and dendritic cells (C4) (CD1C, CLEC10A) (Fig. 3B, C). We then compared the numbers of monocytes, dendritic cells, and individual macrophage subpopulations in different lesions (Fig. 3D). We found that the number of dendritic cells was significantly higher in tumor tissues than in brain metastases, and we also found that macrophage C5 was also significantly higher than in brain metastases (Fig. 3E). Notably, the proportion of macrophage C4 was higher in brain metastases. Moreover, we found that the C4 subpopulation highly expressed cystatin B (CSTB), matrix metalloproteinase 9 (MMP9), chemokine C-C ligand 5 (CCL5), and macrophage migration inhibitory factor (MIF) (Fig. 3F), which have the characteristics of promoting tumor cell migration and invasion. Finally, GSVA results showed that macrophage C4 was mainly enriched in DNA repair, fatty acid metabolism, and oxidative phosphorylation pathways (Fig. 3G). Based on the above results, we hypothesize that macrophage C4 plays an important role in the process of lung cancer brain metastasis.

### Heterogeneity of fibroblasts in different lesions

**Fig. 4:**
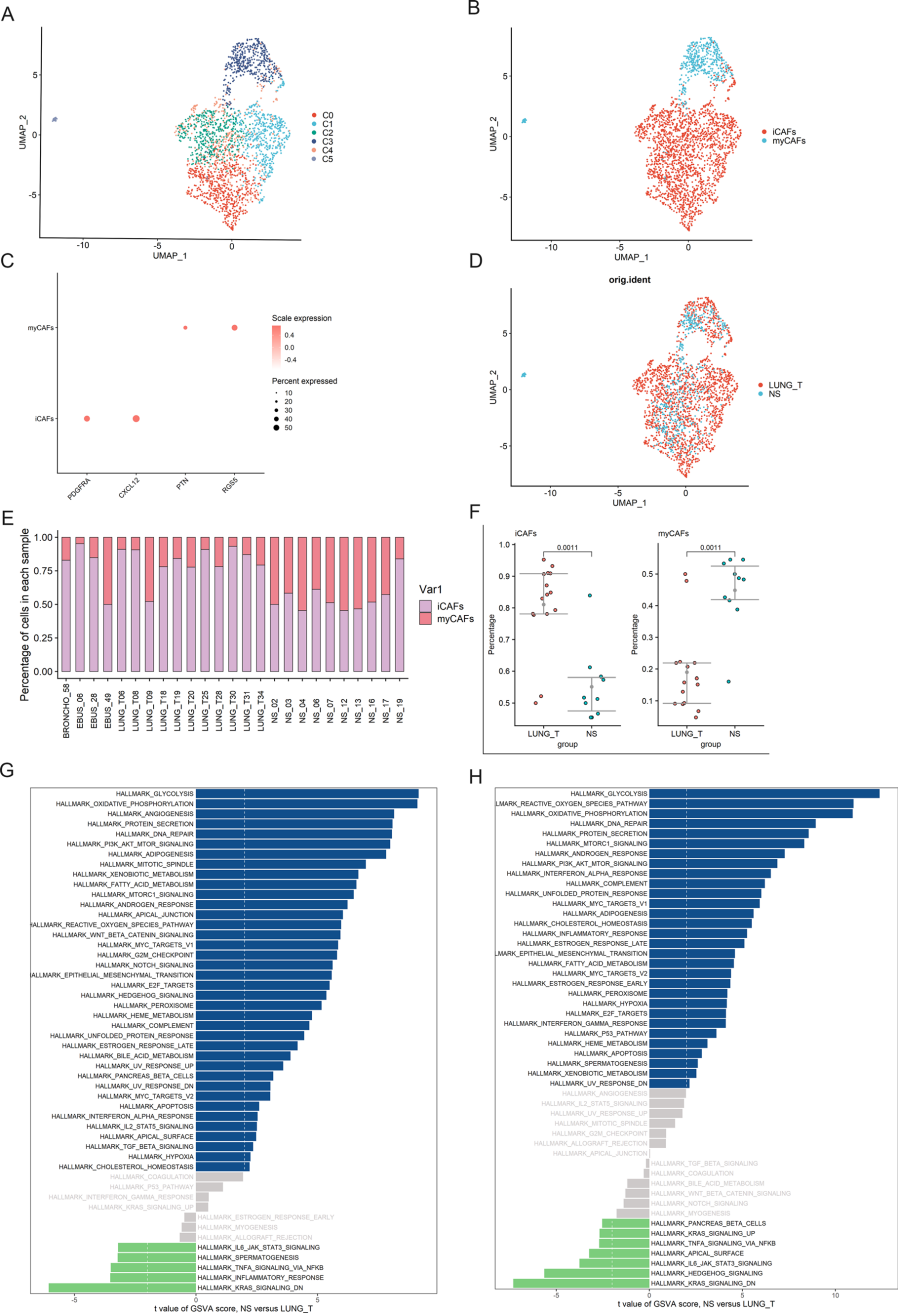
Heterogeneity of fibroblasts in lung adenocarcinoma and brain metastases. **A** UMAP plot shows six subclusters of the fibroblasts. **B** UMAP plot of fibroblasts revealing two subtypes. **C** Canonical cell markers used to identify fibroblasts subtypes. **D** The UMAP plot showing fibroblasts origin. **E** The proportion of fibroblasts subtypes in lung adenocarcinoma and brain metastases. **F** Percentages of each fibroblasts subtype among lung adenocarcinoma and brain metastases. **G** Differences in pathway activities scored per cell by GSVA between lung adenocarcinoma and brain metastases iCAFs. **H** Differences in pathway activities scored per cell by GSVA between lung adenocarcinoma and brain metastases myCAFs.

According to the classical definition of cancer-associated fibroblast (CAF) subtypes, the major subtypes of CAF are myCAF and inflammatory CAF (iCAF). MyCAF is mainly involved in fibrosis, and PTN and RGS5 are marker genes of myCAF. iCAF has greater responsiveness to inflammatory responses and produces large amounts of inflammatory cytokines [20]. As shown in Fig. 4A–D, based on the expression of CAF marker genes, we defined subgroups 0, 1, 2, and 4 as iCAFs and subgroups 3 and 5 as myCAFs. We observed a significant increase of iCAFs in tumor tissues compared with brain metastases, while myCAFs were significantly increased in brain metastases compared with tumor tissues (Fig. 4E, F). By GSVA analysis, we found that iCAFs and myCAFs from brain metastases were highly enriched in pathways that support tumor progression, including glycolysis, oxidative phosphorylation, and EMT, compared with tumor tissues (Fig. 4G, H).

### Upregulation of angiogenic signaling pathways in endothelial cells in brain metastases

**Fig. 5:**
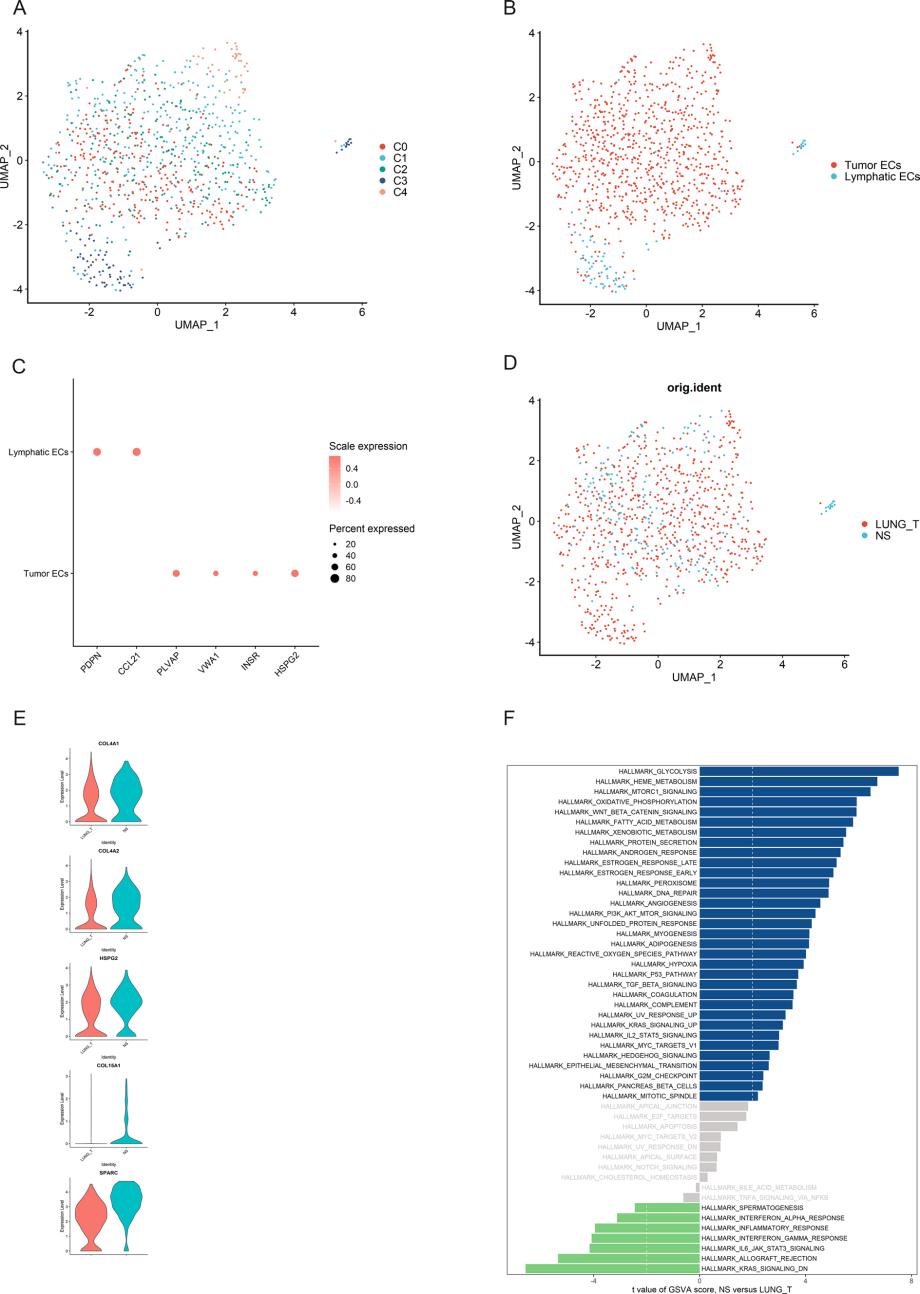
Heterogeneity of endothelial cells in lung adenocarcinoma and brain metastases. **A** UMAP plot shows five subclusters of the endothelial cells. **B** UMAP plot of endothelial cells revealing two subtypes. **C** Canonical cell markers used to identify endothelial cells subtypes. **D** The UMAP plot showing endothelial cells origin. **E** Violin plots of the expression of several marker genes, including COL4A1, COL4A2, HSPG2, COL15A1, and SPARC, in endothelial cells from lung adenocarcinoma and brain metastases. **F** Differences in pathway activities scored per cell by GSVA between lung adenocarcinoma and brain metastases endothelial cells

To describe the landscape of endothelial cells in tumor tissues and brain metastases, we re-clustered 884 endothelial cells (Fig. 5A, D). We obtained five cell subpopulations, including four clusters (C0, C1, C2, C4) of tumor endothelial cells (PLVAP, VWA1, HSPG2, and INSR) and lymphatic endothelial cells (C3) (PDPN, CCL21) (Fig. 5B, C). We found that tumor endothelial cells from brain metastases were highly expressed in angiogenesis-related genes such as COL4A1, COL4A2, HSPG2, COL15A1, and SPARC (Fig. 5E), and GSVA results also showed that tumor endothelial cells from brain metastases were enriched in angiogenesis (Fig. 5F), these results suggest that endothelial cells in brain metastases have a higher angiogenic capacity than primary tumor tissues.

### Epithelial cells in brain metastases have a higher degree of malignancy

**Fig. 6:**
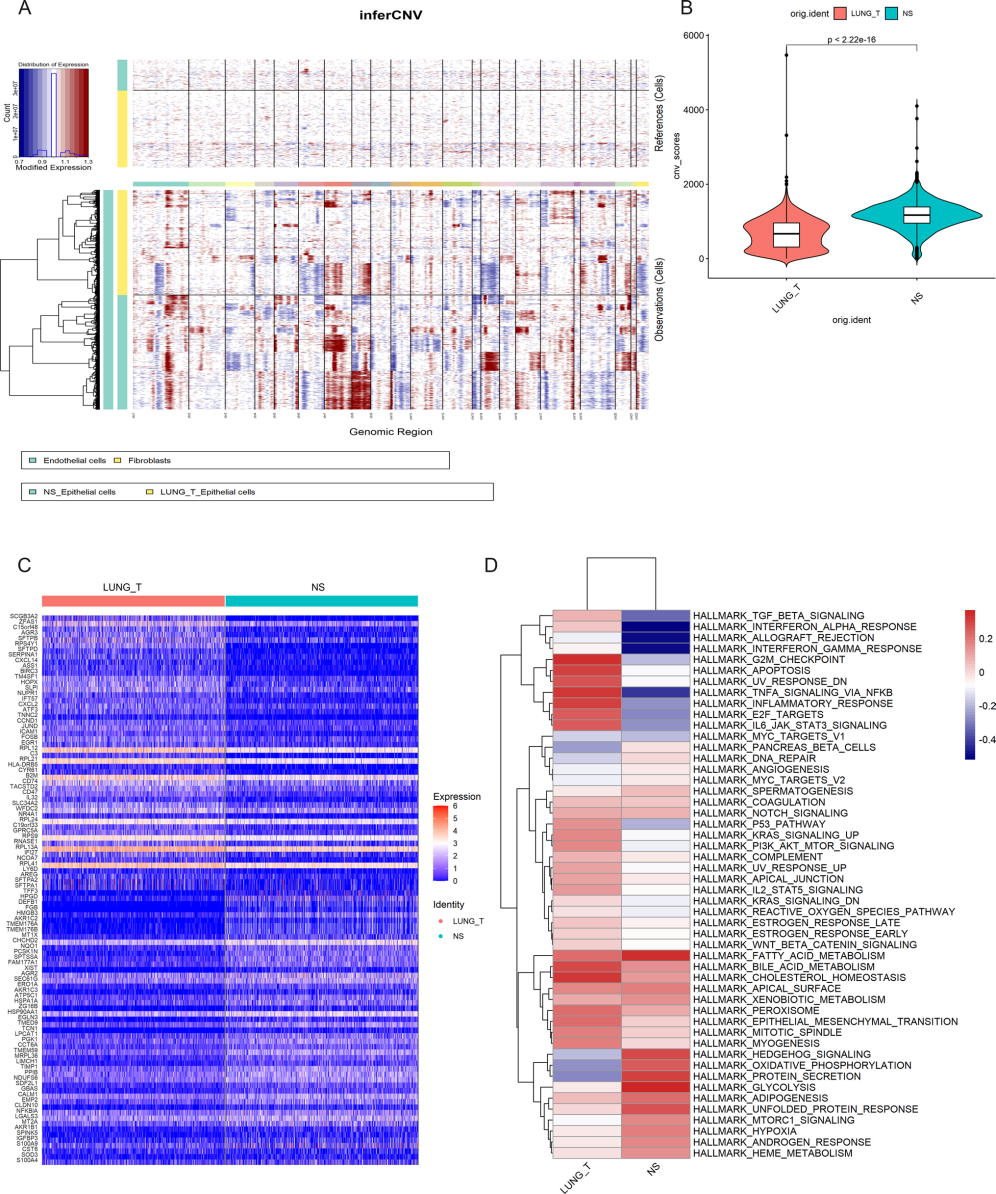
Epithelial cells in brain metastases are more malignant. **A** Copy number variation in Epithelial cells, endothelial cells, and fibroblasts were used as a reference, red represents overexpression of genes, and blue represents low expression. **B** Violin plot indicating the CNV scores of Epithelial cells from lung adenocarcinoma and brain metastases. **C** Heatmap showing the top 50 differential expressed genes between the two groups. **D** Differences in pathway activities by GSVA between lung adenocarcinoma and brain metastases epithelial cells

To analyze the CNV of epithelial cells from different sources, we applied the inferCNV algorithm to analyze the CNV of epithelial cells using the copy number of fibroblasts and endothelial cells in the data as a control. We found extensive CNV in epithelial cells in both tumor tissues and brain metastases (Fig. 6A). Notably, epithelial cells derived from brain metastases had significantly higher CNV levels than epithelial cells from tumor tissues (Fig. 6B). These results suggest to us that multiple copy number variants exist in malignant epithelial cells and that genomic reprogramming occurs in malignant epithelial cells in brain metastases, but the specific mechanisms of alteration still need to be further investigated.

### Analysis of up-regulated genes in epithelial cells from brain metastases by scRNA-seq data

To understand the molecular differences between epithelial cells of different origins, we analyzed up-regulated genes in epithelial cells from brain metastases (Fig. 6C, Supplementary Data 1), which can eliminate the influence of other cells in the tumor microenvironment. Next, we performed the GSVA, which revealed that many metabolic pathways were significantly upregulated in epithelial cells from tumor tissues and brain metastases, including fatty acid metabolism, xenobiotic metabolism, and bile acid metabolism (Fig. 6D). Notably, inflammatory responses as well as interferon alpha response and interferon gamma response pathways were significantly suppressed in epithelial cells in brain metastases compared to tumor tissues. This suggests that the immune function of epithelial cells in brain metastases is more suppressed than in tumor tissue.

### Validation of up-regulated genes in bulk RNA-sequencing data

**Fig. 7:**
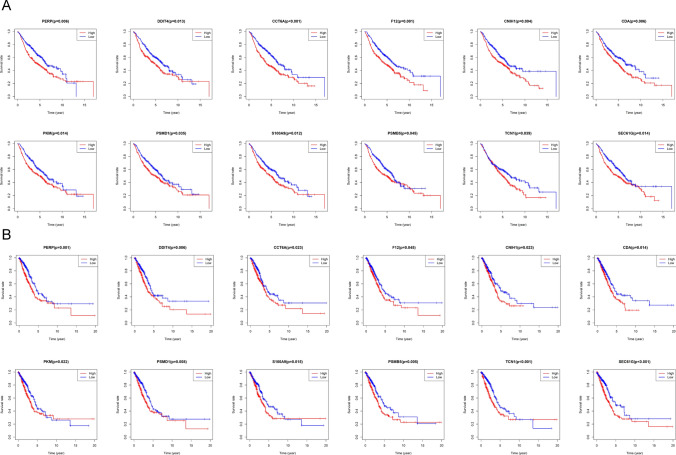
Prognostic role of genes identified by scRNA-seq in TCGA lung adenocarcinoma cohort and GSE68465. **A** Overexpression of genes predicts poor prognosis in TCGA lung adenocarcinoma cohort. **B** Overexpression of genes predicts poor prognosis in GSE68465.

To investigate the impact of these up-regulated genes on the prognosis of lung adenocarcinoma patients, we selected lung adenocarcinoma data from the TCGA and the GSE68465 for prognostic analysis. We found that high expression of twelve genes (Figure S1) simultaneously in these two databases predicted a worse prognosis of lung adenocarcinoma. These twelve genes included PERP, DDIT4, CCT6A, F12, CNIH1, CDA, PKM, PSMD1, S100A9, PSMB5, TCN1 and SEC61G (Fig. 7A, B).

### DDIT4 promotes the proliferation, invasion, and migration of lung cancer cells

**Fig. 8:**
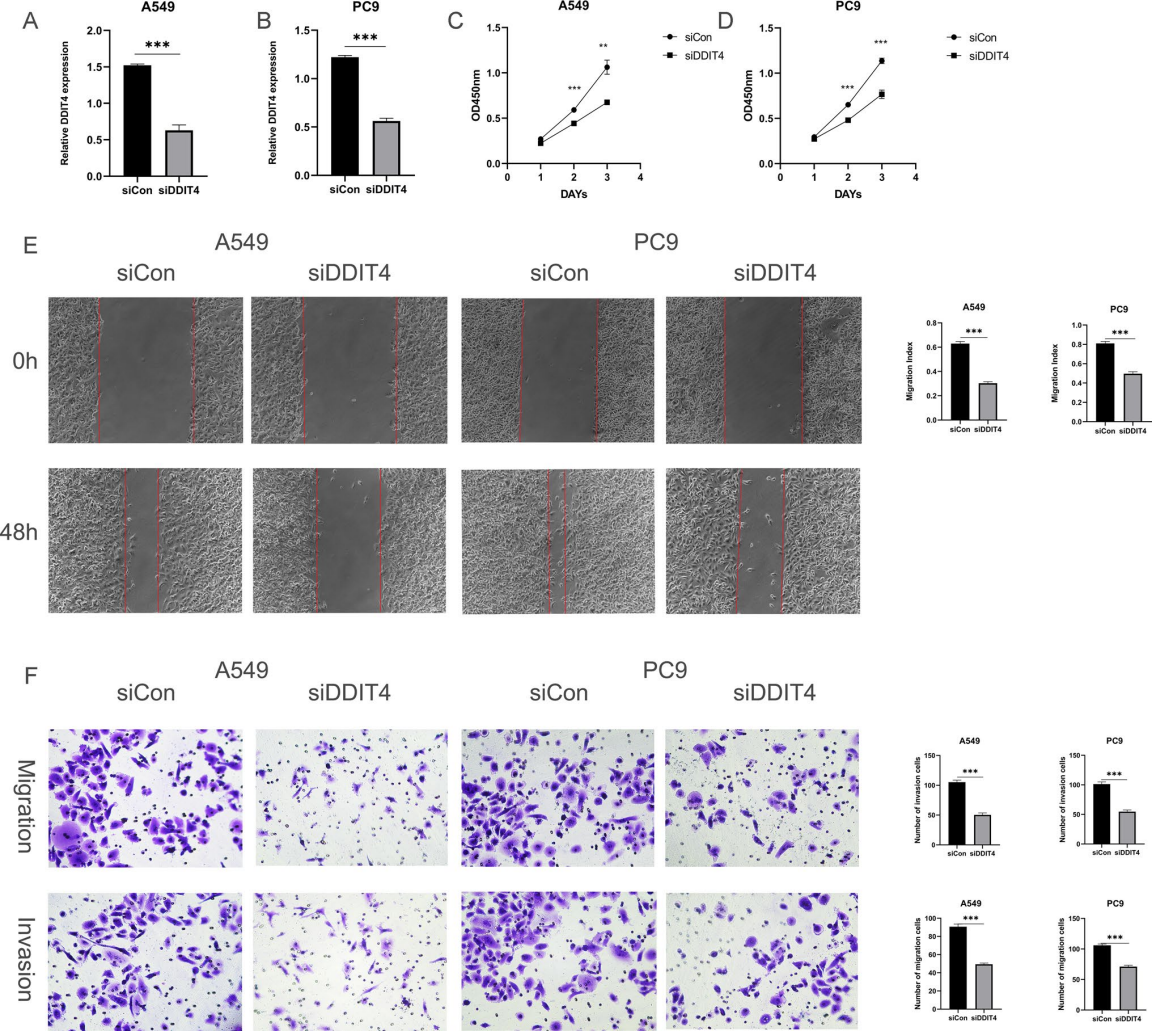
DDIT4 promotes the progression of lung cancer. **A**, **B** The mRNA expression of DDIT4 in A549 and PC9 cells transfected with siRNAs (siDDIT4) or siRNA control (siCon) was measured by qRT-PCR. Means ± SD are shown. ***p < 0.01 by unpaired Student’s t-test. **C**, **D** Downregulation of DDIT4 slowed down A549 and PC9 cell proliferation. **E** siDDIT4 significantly inhibited wound closure compared to the corresponding controls. **F** Migratory and invasive cells were dramatically reduced in A549 and PC9 cells transfected with siDDIT4

Among the twelve genes mentioned above, DDIT4 has been less studied in lung adenocarcinoma, so we chose it for the next study. It has been reported that DDIT4 plays an essential role in tumor progression [21]. In our study, the upregulation of DDIT4 expression was associated with poorer OS in lung adenocarcinoma. Taken together, we hypothesized that DDIT4 plays a key role in the process of lung cancer metastasis. Finally, we found that the downregulation of DDIT4 significantly inhibited the proliferation of lung cancer cells PC9 and A549 (Fig. 8C, D). Furthermore, in wound healing assay, DDIT4 downregulation significantly inhibited wound healing in PC9 and A549 (Fig. 8E). In transwell migration and invasion assays, migration and invasion of cells transfected with DDIT4 were significantly reduced compared to controls (Fig. 8F). Thus, our results revealed that DDIT4 plays a crucial role in the metastasis of lung cancer.

## Discussion

Lung cancer metastases are commonly located in the brain, and there is no effective treatment available, which poses a great challenge for the treatment of patients with brain metastases from lung cancer. Therefore, there is an urgent need to reveal the heterogeneity between lung cancer and brain metastases in the tumor microenvironment with high cellular resolution and the underlying molecular mechanisms of tumor metastasis.

Tumor tissue is mainly composed of tumor cells, immune cells, stromal cells, and extracellular matrix [22]. The presence of immune and stromal cells would greatly affect the sequencing results of tumor cells if analyzed by conventional gene microarrays and transcriptome sequencing. Yet, scRNA-seq can overcome these methodological limitations. By analyzing scRNA-seq, we demonstrated the cellular landscape of lung adenocarcinoma and brain metastases at the single-cell level. In this study, we identified eight cell types from lung adenocarcinoma and brain metastasis samples. Compared with brain metastases, lung adenocarcinoma was enriched in T/NK cells and mast cells. We further analyzed and found that CD8 + T cells in brain metastases showed lower naive scores and higher exhaustion scores, similarly NK cells showed lower naive scores and cytotoxicity scores. This suggests a more severe immune dysregulation in brain metastases. Mast cells are immune cells that accumulate in tumor microenvironments during tumor progression [23]. Mast cells can promote tumorigenesis and tumor progression [24]. The increased accumulation of mast cells in the tumor microenvironment is associated with poor prognosis, increased metastasis, and decreased survival in several cancers, including melanoma [25], prostate cancer [26], and pancreatic ductal adenocarcinoma [27]. Mast cells can release angiogenic mediators, proteases, and growth factors that support tumor development. For example, fibroblast growth factor 2 and vascular endothelial growth factor released by mast cells can trigger a strong angiogenic response [28, 29]. In addition, proteases released by mast cells can activate potential metalloproteinases that promote extracellular matrix degradation, and then promote metastasis [30]. Taken together, we speculate that mast cells play an important role in the progression of lung cancer.

Previous studies have shown that myeloid cells play an essential role in the process of tumor invasion and metastasis [31]. Therefore, we performed a more detailed analysis of the myeloid cells. Our results showed that the number of dendritic cells was significantly higher in tumor tissues than in brain metastases. Surprisingly, we found a higher proportion of macrophage C4 in brain metastatic tissues, and this macrophage subpopulation was highly expressed in genes that promote tumor invasion and metastasis, such as CSTB, MMP9, CCL5, and MIF. For example, a recent study showed that CSTB secreted by macrophages can promote the migration and invasion of tumor cell [32]. In pancreatic cancer, MMP9 secreted by macrophages can promote mesenchymal transition, thereby promoting tumor growth [33]. In prostate cancer, CCL5 derived from macrophages can promote the metastasis of prostate cancer [34]. Finally, in osteosarcoma, MIF promotes the growth and lung metastasis of osteosarcoma by activating RAS/MAPK pathway [35]. Based on the existing research results mentioned above, we speculate that macrophage C4 plays an important role in the brain metastasis of lung cancer, which makes it a potentially valuable target for lung cancer brain metastasis therapy.

CAF is considered to be one of the most abundant stromal cells in almost all solid tumor ecosystems. With the application of scRNA-seq technology, CAF has been shown to be heterogeneous and plastic and to play multiple roles in tumor development, including the promotion of tumor cell proliferation, resistance to therapy and immune rejection, and inhibition of tumor progression [36]. In our study, we found that myCAFs are concentrated in brain metastases. myCAF is a type of CAF, which plays a role in promoting tumor invasion and metastasis in a variety of tumors. For example, in cholangiocarcinoma, myCAF can express heparin-binding epidermal growth factor, which induces the activation of epidermal growth factor receptor in cholangiocarcinoma cells, thus promoting the migration and invasion of tumor cells [37]. In breast cancer, myCAF similarly promotes tumor cell metastasis [38]. Furthermore, in pancreatic and colorectal cancers, myCAF-expressed hyaluronic acid synthetase 2 could promote metastatic tumor growth [39]. By GSVA analysis, we found that myCAF from brain metastases was significantly enriched in some pathways that promote tumor progression, such as glycolysis, oxidative phosphorylation, and EMT. Taken together, we speculate that myCAF plays an important role in brain metastasis of lung cancer.

Angiogenesis is a hallmark of tumors and plays a key role in tumor progression [40]. Blood vessels in tumors provide nutrients and oxygen to the tumor and provide a pathway for tumor metastasis. Recent studies have shown that “vascular secretory factors” released by tumor endothelial cells can promote tumor metastasis. Therefore, in this study, we focused on the differences in tumor endothelial cells between lung adenocarcinoma and brain metastases. We found that tumor endothelial cells from brain metastases highly expressed angiogenesis-related genes, and GSVA results showed that the PI3K/AKT signaling pathway was significantly upregulated in endothelial cells in brain metastases. Previous studies have shown that activation of the PI3K/AKT signaling pathway can stimulate angiogenesis by mediating VEGFR2/VEGFA overexpression [41–43]. GSVA results also showed that tumor endothelial cells from brain metastases were enriched in angiogenesis. The above results suggest that endothelial cells in brain metastases have a stronger angiogenic capacity than endothelial cells in lung adenocarcinoma, which is favorable for lung cancer metastasis.

In tumor cells, gene copy number variation often occurs [44]. We found extensive CNV in epithelial cells in both tumor tissues and brain metastases compared to fibroblasts and endothelial cells. Furthermore, we found that CNV levels were significantly higher in epithelial cells derived from brain metastases than in epithelial cells from tumor tissues. These results suggest that epithelial cells from brain metastases are more malignant than epithelial cells from lung adenocarcinoma. Next, we analyzed the differential expressed genes between the two epithelial cells at the single-cell level and performed a prognostic analysis in combination with the lung adenocarcinoma data from TCGA and GSE68465. Our results found that DDIT4 expression was upregulated in epithelial cells in brain metastases relative to epithelial cells in lung adenocarcinoma and was associated with poor prognosis.

DDIT4, also known as DNA damage response1 and stress-triggered protein, is mainly found in the cytoplasm and nucleus [21]. Compared with normal tissues, high expression of DDIT4 was observed in a variety of tumor tissues, such as colorectal cancer, glioma, head and neck squamous cell carcinoma, and gastric cancer [45–48]. Moreover, upregulation of DDIT4 expression contributes to the reduction of apoptotic processes and promotes proliferation, migration, and invasion of tumor cells in in vitro and in vivo tumor studies. In our study, we associated DDIT4 with the inherent malignant properties of tumors. As a result, we found that DDIT4 promoted the proliferation, migration, and invasion of lung cancer cells. Therefore, DDIT4 could be a potential target for the treatment of lung cancer metastasis.

In this study, we provided a single-cell atlas of lung adenocarcinoma and brain metastases to comprehensively characterize the transcriptomics of lung adenocarcinoma and brain metastases at the single-cell level. We found some results that have not been reported before and require further experimental validation. In addition, we compared advanced and early-stage cancers as the same group of lung adenocarcinoma with brain metastases, which is one of the limitations of this manuscript. Despite these limitations, it can still serve as a valuable resource to identify therapeutic targets for lung cancer brain metastases and to provide personalized treatment decisions for patients with lung cancer brain metastases.

### Supplementary Information

**Supplementary material 1 **

**Supplementary material 2 **

## Data Availability

The datasets supporting the conclusions of this article are included within the article and its additional file.

## Abbreviations

CAF: Cancer-associated fibroblast
CCL5: Chemokine C-C ligand 5
CNV: Copy number variation
CSTB: Cystatin B
DDIT4: DNA damage-inducible transcript 4
FBS: Fetal bovine serum
GEO: Gene expression omnibus
GSVA: Gene set variation analysis
iCAF: Inflammatory CAF
MIF: Macrophage migration inhibitory factor
MMP9: Matrix metalloproteinase 9
MsigDB: Molecular Signatures Database
myCAF: Myofibroblastic CAF
NS: Nervous system
OS: Overall survival
scRNA-seq: Single-cell RNA sequencing
SD: Standard deviation
siRNA: Short interfering RNA
TME: Tumor microenvironment
Treg: Regulatory T cells
UMAP: Uniform manifold approximation and projection

## References

[1] Thai AA, Solomon BJ, Sequist LV, Gainor JF, Heist RS (2021). Lung cancer. Lancet.

[2] Riihimäki M, Hemminki A, Fallah M (2014). Metastatic sites and survival in lung cancer. Lung Cancer.

[3] Page S, Milner-Watts C, Perna M (2020). Systemic treatment of brain metastases in non-small cell lung cancer. Eur J Cancer.

[4] Zhu Y, Cui Y, Zheng X, Zhao Y, Sun G (2022). Small-cell lung cancer brain metastasis: from molecular mechanisms to diagnosis and treatment. Biochim Et Biophys Acta Mol Basis Dis.

[5] Rybarczyk-Kasiuchnicz A, Ramlau R, Stencel K (2021). Treatment of brain metastases of non-small cell lung carcinoma. Int J Mol Sci.

[6] Tiwari A, Trivedi R, Lin SY (2022). Tumor microenvironment: barrier or opportunity towards effective cancer therapy. J Biomed Sci.

[7] Bader JE, Voss K, Rathmell JC (2020). Targeting metabolism to improve the tumor microenvironment for cancer immunotherapy. Mol Cell.

[8] Oshlack A, Robinson MD, Young MD (2010). From RNA-seq reads to differential expression results. Genome Biol.

[9] Li PH, Kong XY, He YZ (2022). Recent developments in application of single-cell RNA sequencing in the tumour immune microenvironment and cancer therapy. Military Med Res.

[10] Lambrechts D, Wauters E, Boeckx B (2018). Phenotype molding of stromal cells in the lung tumor microenvironment. Nat Med.

[11] Guo X, Zhang Y, Zheng L (2018). Global characterization of T cells in non-small-cell lung cancer by single-cell sequencing. Nat Med.

[12] Zilionis R, Engblom C, Pfirschke C (2019). Single-cell transcriptomics of human and mouse lung cancers reveals conserved myeloid populations across individuals and species. Immunity.

[13] Kim N, Kim HK, Lee K (2020). Single-cell RNA sequencing demonstrates the molecular and cellular reprogramming of metastatic lung adenocarcinoma. Nat Commun.

[14] McGinnis CS, Murrow LM, Gartner ZJ, DoubletFinder (2019). Doublet detection in single-cell RNA sequencing data using artificial nearest neighbors. Cell Syst.

[15] Korsunsky I, Millard N, Fan J (2019). Fast, sensitive and accurate integration of single-cell data with Harmony. Nat Methods.

[16] Butler A, Hoffman P, Smibert P, Papalexi E, Satija R (2018). Integrating single-cell transcriptomic data across different conditions, technologies, and species. Nat Biotechnol.

[17] Hänzelmann S, Castelo R, Guinney J (2013). GSVA: gene set variation analysis for microarray and RNA-seq data. BMC Bioinformatics.

[18] Ritchie ME, Phipson B, Wu D (2015). Limma powers differential expression analyses for RNA-sequencing and microarray studies. Nucleic Acids Res.

[19] Patel AP, Tirosh I, Trombetta JJ (2014). Single-cell RNA-seq highlights intratumoral heterogeneity in primary glioblastoma. Science.

[20] Lavie D, Ben-Shmuel A, Erez N, Scherz-Shouval R (2022). Cancer-associated fibroblasts in the single-cell era. Nat cancer.

[21] Tirado-Hurtado I, Fajardo W, Pinto JA (2018). DNA damage inducible transcript 4 gene: the switch of the metabolism as potential target in Cancer. Front Oncol.

[22] Downs-Canner SM, Meier J, Vincent BG, Serody JS (2022). B cell function in the tumor microenvironment. Annu Rev Immunol.

[23] Komi DEA, Redegeld FA (2020). Role of mast cells in shaping the tumor microenvironment. Clin Rev Allergy Immunol.

[24] Ribatti D (2016). Mast cells as therapeutic target in cancer. Eur J Pharmacol.

[25] Ribatti D, Ennas MG, Vacca A (2003). Tumor vascularity and tryptase-positive mast cells correlate with a poor prognosis in melanoma. Eur J Clin Invest.

[26] Nonomura N, Takayama H, Nishimura K (2007). Decreased number of mast cells infiltrating into needle biopsy specimens leads to a better prognosis of prostate cancer. Br J Cancer.

[27] Strouch MJ, Cheon EC, Salabat MR (2010). Crosstalk between mast cells and pancreatic cancer cells contributes to pancreatic tumor progression. Clin Cancer Res.

[28] McHale C, Mohammed Z, Gomez G (2019). Human skin-derived mast cells spontaneously secrete several angiogenesis-related factors. Front Immunol.

[29] Wroblewski M, Bauer R, Cubas Córdova M (2017). Mast cells decrease efficacy of anti-angiogenic therapy by secreting matrix-degrading granzyme B. Nat Commun.

[30] Blair RJ, Meng H, Marchese MJ (1997). Human mast cells stimulate vascular tube formation. Tryptase is a novel, potent angiogenic factor. J Clin Investig.

[31] Christofides A, Strauss L, Yeo A, Cao C, Charest A, Boussiotis VA (2022). The complex role of tumor-infiltrating macrophages. Nat Immunol.

[32] Oelschlaegel D, Weiss Sadan T, Salpeter S (2020). Cathepsin inhibition modulates metabolism and polarization of tumor-associated macrophages. Cancers.

[33] Tekin C, Aberson HL, Waasdorp C (2020). Macrophage-secreted MMP9 induces mesenchymal transition in pancreatic cancer cells via PAR1 activation. Cell Oncol.

[34] Ma J, Shayiti F, Ma J (2021). Tumor-associated macrophage-derived CCL5 promotes chemotherapy resistance and metastasis in prostatic cancer. Cell Biol Int.

[35] Wang C, Zhou X, Li W (2017). Macrophage migration inhibitory factor promotes osteosarcoma growth and lung metastasis through activating the RAS/MAPK pathway. Cancer Lett.

[36] Verginadis II, Avgousti H, Monslow J (2022). A stromal integrated stress response activates perivascular cancer-associated fibroblasts to drive angiogenesis and tumour progression. Nat Cell Biol.

[37] Affo S, Nair A, Brundu F (2021). Promotion of cholangiocarcinoma growth by diverse cancer-associated fibroblast subpopulations. Cancer Cell.

[38] Pelon F, Bourachot B, Kieffer Y (2020). Cancer-associated fibroblast heterogeneity in axillary lymph nodes drives metastases in breast cancer through complementary mechanisms. Nat Commun.

[39] Bhattacharjee S, Hamberger F, Ravichandra A (2021). Tumor restriction by type I collagen opposes tumor-promoting effects of cancer-associated fibroblasts. J Clin Investig.

[40] Lugano R, Ramachandran M, Dimberg A (2020). Tumor angiogenesis: causes, consequences, challenges and opportunities. Cell Mol Life Sci.

[41] Shu X, Zhan PP, Sun LX (2021). BCAT1 activates PI3K/AKT/mTOR pathway and contributes to the angiogenesis and tumorigenicity of gastric cancer. Front Cell Dev Biol.

[42] Li X, Wei Z, Yu H (2021). Secretory autophagy-induced bladder tumour-derived extracellular vesicle secretion promotes angiogenesis by activating the TPX2-mediated phosphorylation of the AURKA-PI3K-AKT axis. Cancer Lett.

[43] Wu Y, Xu X, Liu M (2022). DZW-310, a novel phosphoinositide 3-kinase inhibitor, attenuates the angiogenesis and growth of hepatocellular carcinoma cells via PI3K/AKT/mTOR axis. Biochem Pharmacol.

[44] Erickson A, He M, Berglund E (2022). Spatially resolved clonal copy number alterations in benign and malignant tissue. Nature.

[45] Du F, Sun L, Chu Y (2018). DDIT4 promotes gastric cancer proliferation and tumorigenesis through the p53 and MAPK pathways. Cancer Commun.

[46] Fattahi F, Saeednejad Zanjani L, Habibi Shams Z (2021). High expression of DNA damage-inducible transcript 4 (DDIT4) is associated with advanced pathological features in the patients with colorectal cancer. Sci Rep.

[47] Li W, Hu S, Tian C (2021). TRIP4 transcriptionally activates DDIT4 and subsequent mTOR signaling to promote glioma progression. Free Radic Biol Med.

[48] Zhang Z, Zhu H, Zhao C (2023). DDIT4 promotes malignancy of head and neck squamous cell carcinoma. Mol Carcinog.

