# Supplementary figures and images for: Comparative analysis of single-cell transcriptome reveals heterogeneity in the tumor microenvironment of lung adenocarcinoma and brain metastases
Source: Discov Oncol. 2023 Sep 15;14:174. doi: 10.1007/s12672-023-00784-2 (PMC10504228; doi:10.1007/s12672-023-00784-2)

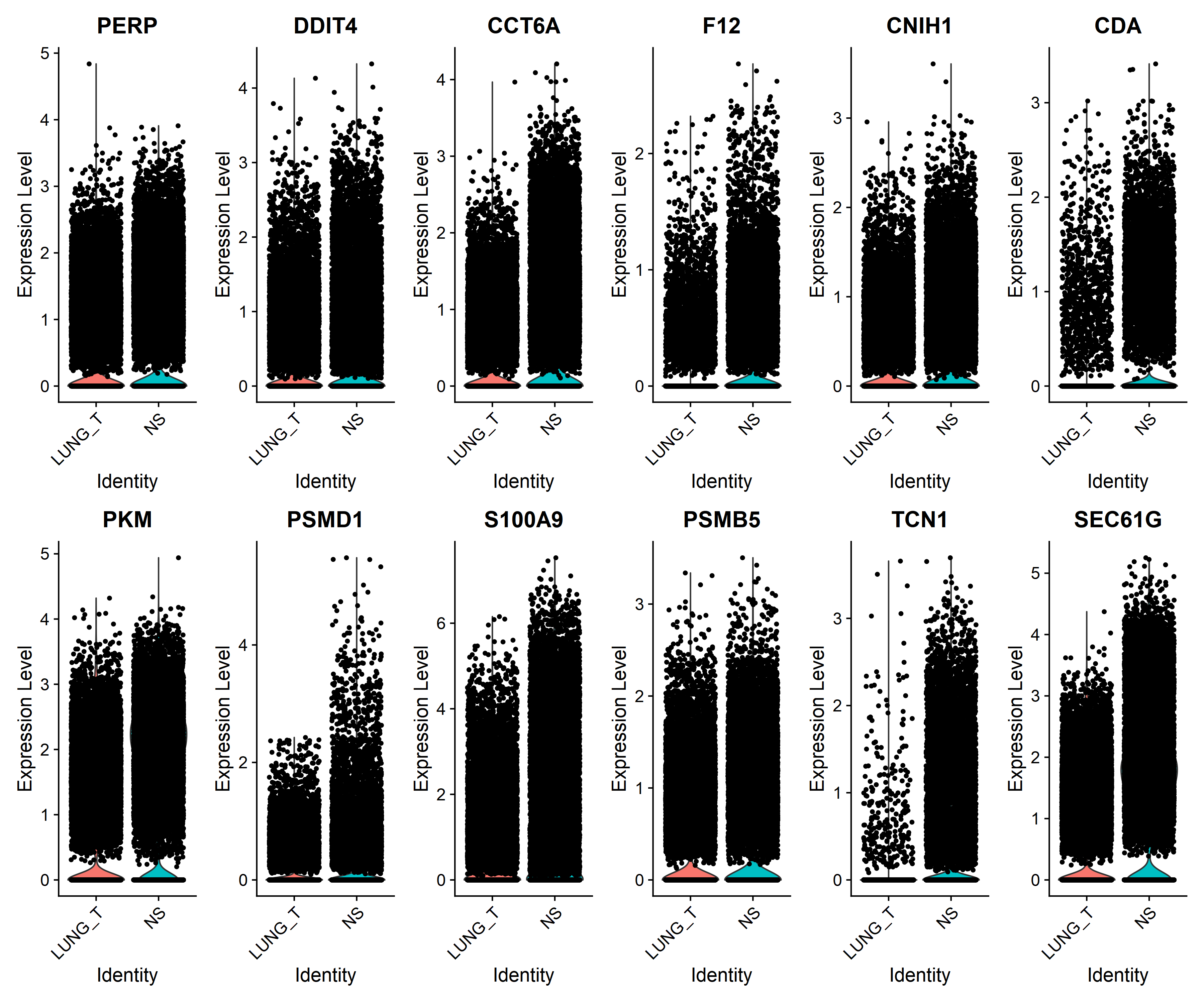

Supplement: Supplementary file 1 — Supplementary material 1 [file 12672_2023_784_MOESM1_ESM.png]
